# Multifocal motor neuropathy presenting as a post-infectious complication of dengue: a CASE report

**DOI:** 10.1186/s12879-018-3334-z

**Published:** 2018-08-22

**Authors:** Rakitha Higgoda, Dilshan Perera, Kanapathipillai Thirumavalavan

**Affiliations:** National Hospital of Sri Lanka, Colombo, Sri Lanka

**Keywords:** Dengue, Multifocal motor neuropathy, Conduction blocks

## Abstract

**Background:**

Dengue infection is an endemic illness in the tropics and it is associated with a wide variety of post infectious complications. With the increasing prevalence of dengue infection in endemic regions, post-infectious neurological complications following dengue infection are now been reported more frequently. We report a patient who developed multifocal motor neuropathy (MMN) with conduction blocks following dengue infection during the immediate post-infectious period. MMN is a rare neurological disorder with an autoimmune etiology and to best of our knowledge this is the first reported case of MMN occurring following dengue infection as a post dengue neurological complication.

**Case presentation:**

A 20 year old Sri Lankan male who was treated for serologically confirmed dengue infection presented to us with 1 month history of bilateral hand weakness which has started 5 days after the dengue infection (5 days after the discharge). On examination he had asymmetrical motor weakness of the hands and to a lesser degree in feet. There was no sensory impairment. Nerve conduction studies confirmed MMN with conduction blocks. He was started on intravenous immunoglobulin therapy for which he showed a good response.

**Conclusion:**

The authors report the first case of MMN complicating dengue fever in a previously healthy male from Sri Lanka**.** Thus it should be borne in mind that although rare, MMN can occur as a post-infectious complication of dengue fever.

## Background

Dengue fever, an acute viral disease transmitted by Aedes mosquitoes, is highly endemic in many tropical and subtropical areas of the world. Sri Lanka suffered its’ largest dengue epidemic in mid-2017 with a resultant significant mortality rate [1]. Dengue infection is well known to be associated and it is associated with a wide variety of post infectious complications. Out of many post-infectious complications of dengue infection, neurological complications have been observed more frequently in the recent past [2, 3].

Depending on the pathogenesis the neurological complications of dengue infection can be categorized into three groups. Firstly, the manifestations due to the neurotropic nature of the virus like meningo-encephalitis and myelitis. Secondly the systemic complications due to the direct effects of active viral infection like ischemic and hemorrhagic strokes, encephalopathy, posterior reversible encephalopathy syndrome, papilloedema, myositis and hypokalemic paralysis. The third category are the post-infectious neurological manifestations like Guillain-Barré syndrome (GBS), Miller-Fisher syndrome, acute disseminated encephalomyelitis (ADEM), encephalomyelitis, cerebellar syndrome, cerebral vasculitis, neuro-ophthalmological disorders (neuromyelitis optica, optic neuritis, maculopathy) and neuritis (oculomotor palsy, abducens nerve palsy, facial nerve palsy, brachial neuritis, phrenic nerve palsy, long thoracic nerve palsy) [3–6].

However, out of diverse range of post-infectious neurological complications of dengue infection, multifocal motor neuropathy has not been previously reported as a post-infectious complication of dengue. We report a patient who was treated for serologically confirmed dengue, developing multifocal motor neuropathy with conduction blocks 5 days after recovering from dengue infection.

Multifocal motor neuropathy (MMN) with conduction blocks (CB) is an acquired immune-mediated demyelinating neuropathy with slowly progressive weakness of distal limbs (mainly upper limbs) [7]. Although post-infectious etiology is not commonly entertained in the pathogenesis of MMN, it is possible that the pathogenesis of MMN in this patient was related to a post dengue immune mediated mechanism.

## Case presentation

A 20 year old Sri Lankan male who was employed as a helper in a grocery, admitted to our unit with weakness of both hands of 1 month’s duration. He was treated for serologically confirmed (Dengue NS1 antigen positive) dengue fever approximately 5 weeks ago at the local hospital and had made an uneventful recovery. He has been given 5 days of inward treatment and the records from the local hospital revealed that he had simple dengue fever with no evidence of fluid leakage.

Five days after discharge from the hospital he has first noticed the weakness of his right hand when he dropped a glass of water due to poor grip. Weakness was more in the right hand which was his dominant hand and it was slowly progressive over 1 month. At the time of presentation to us he could not write or button on his shirt due the weakness of the hands. Weakness of the left hand was milder than that of the right. The weakness was confined to hands and did not involve forearms or arms. He denied any accompanying numbness, parasthesia or pain.

On inquiry he admitted that there was slight weakness of both feet which did not significantly interfere with walking. There was no associated neck/back pain or bladder/bowel incontinence. He did not complain of difficulty in breathing, diplopia, dysphagia, nasal regurgitation, dysarthria or fatigability. He did not give a recent history of trauma to the spine/neck or any preceding diarrheal illness or skin rash.

He had no previously diagnosed long term medical ailments and has not undergone any surgical procedures in the past. He was not on any long term medications and he denied smoking, use of alcohol or illicit drugs. He did not give a family history of any progressive neurological conditions.

On general examination he had an average built with no pallor, lymphadenopathy or any signs of malnutrition. No skin rashes or hypopigmented patches were noted. There was minimal small muscle wasting of bilateral hands and feet. No muscle fasciculations were noted. Distal upper limb (hand) power was diminished asymmetrically, right hand demonstrating a power of 3 out of 5 and left hand demonstrating a power of 4 out of 5. All fine finger movements including flexion, extension, abduction and adduction were affected with some degree of weakness in wrist extension as well. Bilateral supinator and biceps reflexes were diminished.

Distal lower limb (feet) power was also diminished but was less pronounced (power grade 4) when compared to the degree of hand weakness. Bilateral foot dorsiflexion was weak. Ankle jerks were elicited with reinforcement whereas the knee jerks were elicited without reinforcement. There was no objective sensory impairment of touch, pain, temperature, vibration and joint position sensations in both upper and lower limbs. Bilateral plantar responses were down going. No palpable nerve thickening identified. No cerebellar signs were demonstrated and his gait showed a minor degree of high stepping due to weak dorsiflexion. Examination of higher functions and cranial nerves including the fundal examination revealed no abnormality.

Examination of the cardiovascular, respiratory systems and the abdomen was essentially normal.

### Investigations revealed the following results

Full blood count revealed white blood cell count: 8.5 × 10^9^/L, platelet count: 274 × 10^9^/L, hemoglobin 12 g/dl with normal red cell indices. Blood picture showed normochromic normocytic cells with some reactive lymphocytes suggestive of a recent viral infection. Serum creatinine 80 μmol/l (60 - 110 μmol/l), serum sodium 138 mmol/l (135 - 145 mmol/l), serum potassium 3.8 mmol/l (3.5 - 5 mmol/l), serum magnesium 0.9 mmol/l (0.8–1.1 mmol/l), serum ionized calcium 1.2 mmol/l (1.05–1.30 mmol*/*l). Liver profile: AST 21u/l (10 - 40u/l), ALT 13u/l (7–56 u/l), ALP 67u/l (100–360 u/l), serum total bilirubin 0.7 mg/dl (0.1–1.2 mg/dl), serum albumin 36 g/l (35 - 50 g/l), serum globulin 32 g/l (20 - 35 g/l). CPK levels were normal. Inflammatory markers: ESR 25 mm/hour and CRP < 6 mg/dl.

**Fig. 1:**
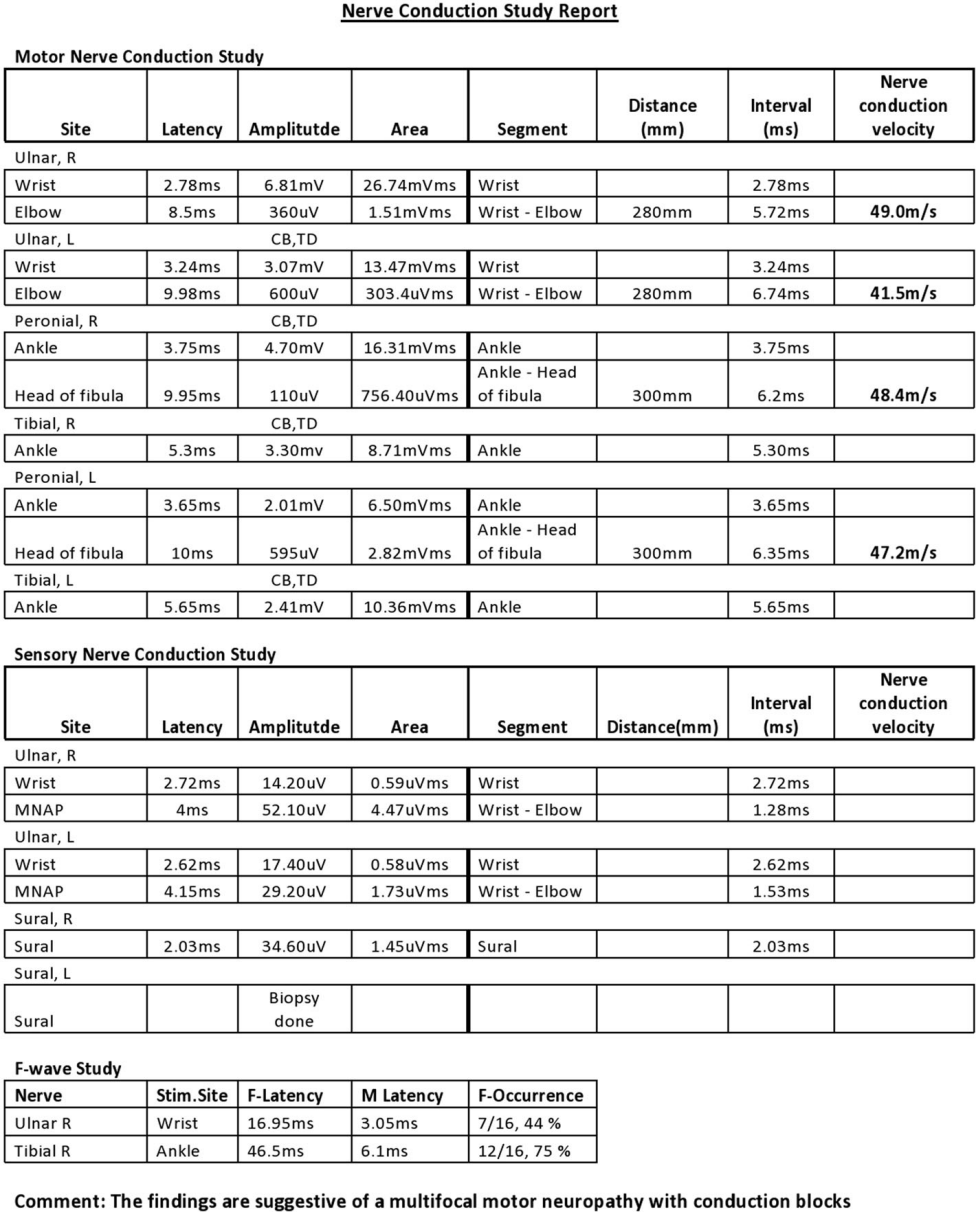
Findings of nerve conduction test of upper and lower limbs

Nerve conduction study revealed findings in keeping with multifocal motor neuropathy with conduction blocks involving the distal upper and lower limb peripheral nerves without any conduction abnormalities in the sensory nerves (Fig. 1).

CSF analysis did not show any increase in proteins or cells and the values were within the normal limits. Anti-GM1 IgM antibody test was not carried out due to the high cost of the test and the patient’s unstable financial background. A sural nerve biopsy (a sensory nerve) was carried out and revealed histologically unremarkable nerve fibres and blood vessels with no evidence of inflammation, atrophy or granulomata formation. Recent dengue infection was confirmed with positive dengue IgM and IgG antibodies with enzyme-linked immunosorbent assay (ELISA).

As the patient fulfilled criteria, the diagnosis of multifocal motor neuropathy with conduction blocks was confirmed. He was then referred to the neurologist and was started on intravenous immunoglobulin (IVIg) therapy (2 g/kg/day) which was given for 5 days. He showed a mild improvement of his neurological weakness with the treatment and outpatient physiotherapy was arranged. The next immunoglobulin dose was planned to be given after 2 weeks.

## Discussion and conclusions

Dengue infection is seen in increasing numbers in Sri Lanka and in fact the largest number of dengue cases is being reported in 2017 [1]. MMN on the other hand is a very rare neurological disease of peripheral motor nerves with a prevalence of 1 to 2 per 100,000 [8].

The three clinical and electro-diagnostic criteria for a definitive diagnosis of MMN are presence of isolated motor weakness (without a sensory loss) in the distribution of two or more nerves for more than a month, presence of a definite motor conduction block in at least one motor nerve with intact sensory nerve conduction and absence of exclusion criteria. There are four exclusion criteria. First, the presence of upper motor nerve lesion signs like hyper-reflexia, increased tone, extensor plantar response or clonus. Diffuse symmetric weakness of the involved limbs early in the course of the illness is also considered as one of the exclusion criteria. The other two criteria are significant sensory involvement (apart from a minor loss of vibration sense in the lower limbs) and significant weakness of bulbar muscles [9, 10].

Our patient’s clinical and electro-diagnostic findings were in keeping with the above mentioned diagnostic criteria for MMN.

Interestingly about half of the patients diagnosed with MMN demonstrate IgM antibodies against the ganglioside GM1 (anti GM1) in their serum [11]. This reinforces the fact that the pathogenesis of MMN is immune mediated. GM1 plays a major role in neuronal signal conduction being present in high concentrations at Ranvier and para-nodes of myelin sheaths of the peripheral nerves [12]. This particular antibody is also found in patients with similar immune-mediated neurological disorders like acute motor axonal neuropathy (AMAN) variant of GBS [13, 14]. We were unable to carry out the test in our patient due to financial constraints.

However a post infectious mechanism driving the immune mediated process in MMN is not very well established. Apart from the idiopathic form of MMN, the only well known cause of MMN is post infliximab therapy which is implicated in pathogenesis of a severe form of MMN [15–17]. However GBS, a similar peripheral demyelinating neuropathy is well known to be associated with a post dengue immune mediated etiology [3]. It is thought that the host immune system mounts an immune response against the dengue virus which produces antibodies that cross react with components of myelin in peripheral nerves by which it causes GBS as a post-infectious complication. This phenomenon is known as molecular mimicry [18].

In our case MMN occurring approximately a week after dengue infection strongly queries a post-infectious immune mediated process in pathogenesis of MMN. However we also admit the possibility of the dengue infection and the following MMN being two independent events occurring coincidently. But the immune mediated nature of MMN and the association of dengue fever with other similar post infectious neurological conditions strongly suggest that MMN in this patient was related to the preceding dengue infection.

On the other hand dengue fever is proved to be associated with widespread post infectious neurological complications which include both central and peripheral nervous system manifestations as described above [2, 3]. It is theoretically possible that post dengue MMN occurring in a similar mechanism as seen in post dengue GBS. Further studies exploring this aspect will aid in elucidating the possible post infectious mechanisms in pathogenesis of MMN. But the rarity of the MMN might make it practically difficult.

The mainstay of treatment of MMN is intravenous immune-globulin (IVIg). It has been able to bring about a satisfactory response in up to 80% of the patients and sometimes the improvement in motor weakness is seen as early as the first week of therapy. IVIg is thought to act by inhibiting the complement deposition at Ranvier nodes of peripheral nerves [19, 20]. In our case prompt administration of IVIg resulted in some degree of improvement of the muscle weakness within 2 weeks of therapy. Early diagnosis of the condition is crucial as delay in initiation of treatment may reduce the chances of recovery.

This case report illustrates that MMN, although rare, can occur as a complication of dengue fever and to best of our knowledge it is the first such case to be reported to date. In an era in which dengue infection is occuring in epidemics, it is important to keep in mind the possibility of such post infectious neurological conditions complicating the picture. It also adds to the variety of post-infectious neurological conditions known to occur following dengue infection.

## Abbreviations

ALP: Alkaline phosphatase
ALT: Alanine aminotransferase
AST: Aspartate aminotransferase
CB: Conduction blocks
CPK: Creatine phosphokinase
CRP: C- reactive protein
ESR: Erythrocyte sedimentation rate
GBS: Guillain-Barré syndrome
IVIg: Intravenous immune globulin
MMN: Multifocal motor neuropathy

## References

[1] National Dengue control unit of Sri Lanka http://203.143.20.230/dengue.health.gov.lk/public_html/index.php/information-on-dengue/sri-lankan-situation. Accessed 20 October 2017.

[2] Verma R, Sharma P, Garg RK, Atam V (2011). Neurological complications of dengue fever: experience from a tertiary center of North India. Ann Indian Acad Neurol.

[3] Murthy JM (2010). Neurological complications of dengue infection. Neurol India.

[4] Li GH, Ning ZJ, Liu YM, Li XH (2017). Neurological manifestations of dengue infection. Front Cell Infect Microbiol.

[5] Mai N, Phu N, Nghia H (2018). Dengue-associated posterior reversible encephalopathy syndrome, Vietnam. Emerg Infect Dis.

[6] Herath HM, Hewavithana JS, De Silva CM (2018). Cerebral vasculitis and lateral rectus palsy – two rare central nervous system complications of dengue fever: two case reports and review of the literature. J Med Case Rep.

[7] Nagale SV, Bosch EP (2003). Multifocal motor neuropathy with conduction block: current issues in diagnosis and treatment. Semin Neurol.

[8] Chaudhry V, Swash M (2006). Multifocal motor neuropathy: is conduction block essential?. Neurology.

[9] European Federation of Neurological Societies (2010). Peripheral nerve society guideline on management of multifocal motor neuropathy. Report of a joint task force of the European Federation of Neurological Societies and the peripheral nerve society-first revision. J Peripher Nerv Syst.

[10] Bromberg MB, Franssen H (2015). Practical rules for electrodiagnosis in suspected multifocal motor neuropathy. J Clin Neuromuscul Dis.

[11] Nobile-Orazio E, Giannotta C, Musset L, Messina P, Léger JM (2014). Sensitivity and predictive value of anti-GM1/galactocerebroside IgM antibodies in multifocal motor neuropathy. J Neurol Neurosurg Psychiatry.

[12] Willison HJ, Yuki N (2002). Peripheral neuropathies and anti-glycolipid antibodies. Brain.

[13] Van Sorge NM, Yuki N, Jansen MD (2007). Leukocyte and complement activation by GM1-specific antibodies is associated with acute motor axonal neuropathy in rabbits. J Neuroimmunol.

[14] Susuki K, Rasband MN, Tohyama K (2007). Anti-GM1 antibodies cause complement-mediated disruption of sodium channel clusters in peripheral motor nerve fibers. J Neurosci.

[15] Cocito D, Bergamasco B, Tavella A (2005). Multifocal motor neuropathy during treatment with infliximab. J Peripher Nerv Syst.

[16] Rodriguez-Escalera C, Belzunegui J, Lopez-Dominguez L, Gonzalez C, Figueroa M (2005). Multifocal motor neuropathy with conduction block in a patient with rheumatoid arthritis on infliximab therapy. Rheumatology (Oxford).

[17] Singer OC, Otto B, Steinmetz H, Ziemann U (2004). Acute neuropathy with multiple conduction blocks after TNFalpha monoclonal antibody therapy. Neurology.

[18] Hahn AF (1998). Guillain-Barre syndrome. Lancet.

[19] Nguyen TP, Chaudhry V (2011). Multifocal motor neuropathy. Neurol India.

[20] Yuki N, Watanabe H, Nakajima T, Spath PJ (2011). IVIG blocks complement deposition mediated by anti-GM1 antibodies in multifocal motor neuropathy. J Neurol Neurosurg Psychiatry.

